# Function and autophagy of monocyte-derived dendritic cells is affected by hepatitis B virus infection

**DOI:** 10.1186/s12865-023-00571-2

**Published:** 2023-09-26

**Authors:** Hua Xu, Juan Kang, Shan Zhong, Min Chen, Peng Hu, Hong Ren, Zhi Zhou, Yu Lei

**Affiliations:** 1 Department of Infectious Diseases, Key Laboratory of Molecular Biology for Infectious Diseases (Ministry of Education), Institute for Viral Hepatitis, the Second Affiliated Hospital, Chongqing Medical University, No.288 Tianwen Rd., Nan Ping District, Chongqing, 400060 People’s Republic of China; 2 Department of oncology, Chongqing Hospital of Traditional Chinese Medicine, No. 6, 7 Branch Road, Panxi, Jiangbei District, Chongqing, 400021 China

**Keywords:** Hepatitis B virus, Monocyte-derived dendritic cell, Phenotype, Cytokine, Autophagy

## Abstract

**Background:**

The role of dendritic cells and the autophagy state of dendritic cells in the immune response of hepatitis B virus (HBV) infection was still controversial. In this study, we carefully examined the phenotype, function and autophagy pathway of dendritic cells in HBV infection.

**Methods:**

Monocyte-derived dendritic cells from healthy blood donors and patients with chronic HBV infection were stimulated by lipopolysaccharide, supernatant of HepG2.2.15 cells or supernatant of HepG2 cells respectively. Phenotype of dendritic cells was examined by flow cytometry and cytokines secretion was detected by enzyme-linked immunosorbent assay. Autophagy related proteins were detected by western blot and immunofluorescence analysis.

**Results:**

Our results showed that the expression of both major histocompatibility complex II molecules and co-stimulated molecules including cluster of differentiation antigen 80, cluster of differentiation antigen 86 in the monocyte-derived dendritic cells from patients with chronic HBV infection was significantly higher than that from healthy donors when cultured with supernatant of HepG2.2.15 cells. The amount of cytokines, including tumour necrosis factor-α, interleukin-10 and interleukin-12, secreted by monocyte-derived dendritic cells from patients with chronic HBV infection was also significantly higher than that from healthy donors when stimulate by HBV. Interestingly, the expression level of autophagy-related proteins including autophagy-related protein5 and associated protein 1 light chain in dendritic cells from patients with chronic HBV infection was significantly increased when compared with that from healthy donors when re-exposed to HBV.

**Conclusions:**

Our results indicated that dendritic cells from patients with chronic HBV infection could intensively present antigen and express co-stimulatory molecules. The increased activation of dendritic cells might be related to the enhanced autophagy of dendritic cells in HBV infection.

**Supplementary Information:**

The online version contains supplementary material available at 10.1186/s12865-023-00571-2.

## Background

Hepatitis B virus (HBV) is a hepatotrotic member of the hepadnavirus family that causes acute and chronic hepatitis, cirrhosis and hepatocellular carcinoma (HCC) [1–3]. However, HBV is noncytopathic for hepatocytes and the outcome of HBV infection is mainly dependent on the immune-mediated host-virus interaction. [4]. During acute HBV infection, in patients who successfully clear the virus, a multispecific and polyclonal cytotoxic T lymphocytes (CTL) response and a strong type-1 T helper cell response was exhibited [5–7]. However, in patients with chronic HBV infection (CHB), the HBV-specific T lymphocytes is generally undetectable. These findings suggests that HBV-specific T lymphocytes dysfunction might cause viral infection persistence [8, 9]. Series of studies showed that in persistent HBV infection, the maturation and function of DCsare severely impaired, that could not activate the efficient HBV-specific immune response for virus clearance. This might be the reason of immune tolerance state of CHB [10–13]. In contrast, several other researches found that the frequency and phenotype of DCs were similar between HBV patients and healthy donors (healthy donors) [14, 15]. Whether DCs in patients with CHBare phenotypically and functionally equal to DCs from healthy controls has been a longstanding point of debate.

DCs are the most professional antigen presenting cells (APCs) in the body [16]. When encountering pathogens, DCs internalize, process and present antigens, then migrate to T lymphocytes-rich areas of lymphoid organs and gradually become maturation during migration. Mature DCs stimulate T lymphocytes for activation and differentiation through the expression of major histocompatibility complex (MHC), co-stimulatory molecules and the production series of cytokines. Autophagy is known as the process that cell digest portions of their interiors to recycle nutrients, remodel and dispose of unwanted cytoplasmic constituents, which also plays an important role in virus deletion and arousing the immune response. A number of researches have showed that the autophagy was important for the modulation of DCs functions. DCs processing foreign antigens require high levels of endocytic and lysosomal activity that are tightly linked to autophagy. Autophagy machinery was also involved in the migration and production of inflammatory cytokines of DCs, as well as in activating T lymphocytes responses by optimizing antigen processing and both MHC-II and MHC-I presentation. [17–20]. Recent researches about autophagy in HBV infection demonstrated that the autophagy machinery is needed for HBV production and envelopment in the hepatocytes both in the HBV transgenic mice and patients with HBV infection [21–24]. An increased amount of autophagic vacuoles including autophagosomes and autolysosomes were shows in hepatocytes in which HBV productively replicated. However, during the persistent HBV infection, whether the status of autophagy in DCs is affected is still unknown. Whether the phenotype and function of DCs in CHBis associated with the presumed alteration of autophagy is also unclear.

In this study, we carefully investigated the phenotypes and functions of blood monocyte-derived dendritic cells (mo-DCs) in patients with CHBand health controls. We also examined the expression of autophagy related genes and proteins states in mo-DCs which exposed to HBV and explored the correlation among autophagy, DCs maturation and HBV infection.

## Methods

### Study subjects

In this study, we chose patients with CHB whose hepatitis B virus surface antigen (HBsAg) was positive for ≥ 6 months and the serum HBV DNA concentration ≥ 10^4^ IU/ml, had normal liver function (alanine aminotransferase [ALT] level of <40 U/L, aspartate aminotransferase [AST] level of <30 U/L). Patients were excluded if they were coinfected with other hepatitis viruses or HIV or had received antiviral or immunomodulatory treatment before blood sampling. Patients with primary biliary cholangitis, primary hepatocellular carcinoma were also excluded. All serological markers for HAV, HBV, HCV, HEV and HIV were negative in healthy controls. The HD had no other forms of liver diseases, including autoimmune or alcoholic liver disease, drug hepatitis or Wilson’s disease and had no severe diseases in other system, all healthy blood donors were not vaccinated against hepatitis B. The patients’ demographic characteristics are summarized in supplementary Table 1.

These studies were conducted according to the Declaration of Helsinki guidelines, and were approved by the Ethical Committee of Second Affiliated Hospital of Chongqing Medical University. Written informed consent was obtained from all participants.

## HepG2.2.15-derived HBV

HepG2 cells, a non-HBV-containing cell line, were established from human hepatoblastoma, and hepG2.2.15 cells are derived from HepG2 and characterized by having stable HBV expression and replication in the culture system. HepG2.2.15 cells and HepG2 cells which be kept by our laboratory were cultured. Supernatant was collected until the cells stably grew and passage and HBV DNA levels in the supernatant were measured in the Clinical Chemistry Laboratory at the Second Affiliated Hospital of Chongqing Medical University. The supernatant of HepG2.2.15 with the HBV DNA level above 10^4^ IU/ml was used.

### Generation of mo-DCs

Peripheral blood mononuclear cell (PBMC) from patients with CHB and HDwere isolated by density gradient separation using percoll centrifugation (Solarbio). cluster of differentiation antigen14^+^ (CD14^+^) monocytes were purified by using anti-CD14 mAb-conjugated magnetic beads (Miltenyi Biotec). Sorted cells with more than 90% purity were used. Purified monocytes were then cultured with 200 ng/ml recombinant human granulocyte-macrophagecolony stimulating factor (GM-CSF) plus 200 ng/ml interleukin-4 (IL-4) (PeproTech) for 6 days and were called monocyte-derived (Mo-DCs). A half of the culture medium was replaced by fresh medium containing GM-CSF and IL-4 on day 3. On day 6, cultured cells were exposed to RPMI 1640 (blank group), RPMI 1640 with 10 ug/ml lipopolysaccharide (LPS) (LPS group), supernatant of HepG2.2.15 cells (HBV group) and supernatant of HepG2 cells (HepG2 group) until day 10.

### Flow cytometry analysis

Newly isolated monocytes or cultured cells were harvested and incubated with appropriate dilutions of the following fluorochrome-conjugated antibodies: CD14-PE-Cy5.5 (BD Biosciences, New Jersey, USA), CD11c-FITC (BD Biosciences, New Jersey, USA), Propidium Iodide [PI] (Beyotime, Shanghai, China), (human leukocyte antigen- DR-PE)HLA-DR-PE (BD Biosciences, New Jersey, USA), CD123-APC (BD Biosciences, New Jersey, USA), CD80-PE-Cy7 (BD Biosciences, New Jersey, USA), CD83-PE-Cy7 (BD Biosciences, New Jersey, USA), CD86-PE-Cy7 (BD Biosciences, New Jersey, USA). Isotype-matched control antibodies (BecktonDickinson, San Jose, USA) were used to correct nonspecific binding. Then the stained cells were analysed using a FACS Canto II cytometer and FACSDiva software (version4.1; Becton Dickinson) or FlowJo analysis software (Tree Star, Ashland, OR, USA).

### Cytokines detection

Levels of plasma cytokines, including tumour necrosis factor-α (TNF-α)and IL-10, were determined by enzyme-linked immunosorbent assay (ELISA) using commercial human ELISA Kit from Biosource International (Nivelles, Belgium) and IL-12p70 was determined using an ELISA kit from Diaclone (Besancon, France) in accordance with the manufacturer’s instructions.

### Western blot analysis

Proteins from cultured cells were collected and the concentration of proteins was determined using the BCA Protein Assay Kit (Beyotime, Shanghai, China). Then proteins were incubated primary antibodies, including associated protein 1 light chain (LC3)A/B RabMAb (Epitomics, California, USA), Agt5 RabMAb (Epitomics, California, USA), beclin-1 RabMAb (Epitomics, California, USA), followed by peroxidase-conjugated goat anti-rabbit IgG (Sigma, Missouri, United States). The blots were visualized using a western blotting luminol reagent system (iNtRON Biotechnology) and autoradiography.

### Immunofluorescence analysis

Cells were seeded in 24-well plates (5 × 10^5^/well) on cover slips and were fixed with − 20℃ Acetone. The fixed cells were incubated with primary antibodies against LC3A/B RabMAb (Epitomics, Missouri, United States), and then incubated with Alexa488-conjugated secondary IgG (MultiSciences (Lianke) Biotech, Hangzhou, China). Following those incubation, the nuclei were stained with Propidium Iodide [PI] (Beyotime, Shanghai, China). Images were analyzed with a Nikon confocal laser-scanning microscope and Nikon Confocal software version (NIS-Elements Viewer 4.2).

### Statistical analysis

All data were analyzed using SPSS 13.0 software (SPSS Inc, Chicago, IL, USA). Data were expressed as mean ± SD or mean ± SE. One-way ANOVA was used for comparisons between groups. The t test was used for two independent data. A two tailed P < 0.05 was considered statistically significant. Images were performed on GraphPad prism 5 Demo.

## Results

### CD14^+^ monocytes could be developed to DCs after exposed to HBV

**Fig. 1:**
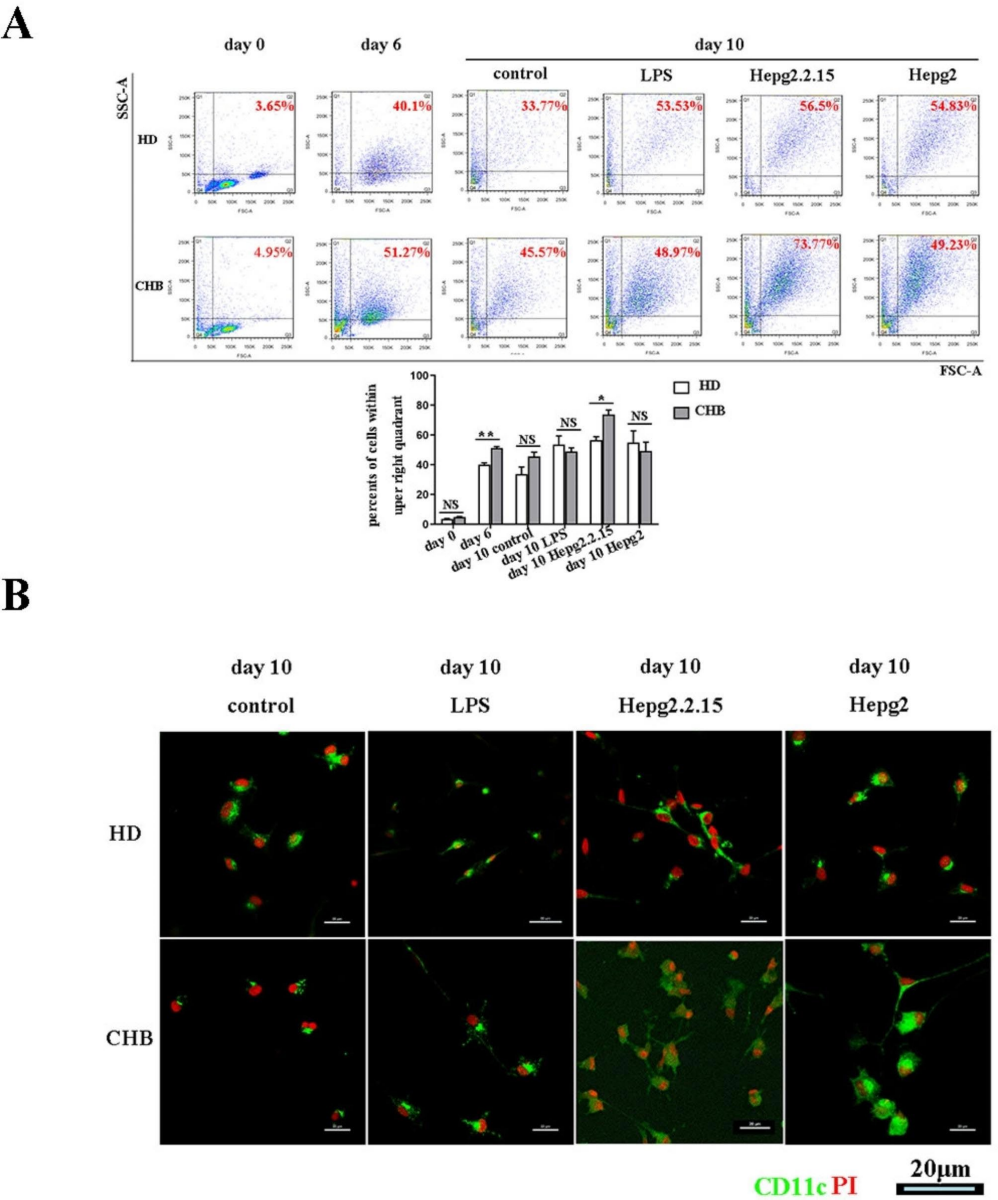
Morphology of Monocyte-derived dendritic cells (Mo-DCs) in culture. At day 0 (Day 0), cluster of differentiation antigen14^+^ (CD14^+^) monocytes from health donors (HD) and patients with chronic HBV infection (CHB) were isolated and incubated with recombinant human granulocyte-macrophagecolony stimulating factor (GM-CSF) plus interleukin-4(IL-4)for 6 days (Day 6) and so called Mo-DCs. Then the cells were continued to be cultured with unstimulated condition (control group), lipopolysaccharide (LPS), supernatant of HepG2 cells and supernatant of HepG2.2.15 cells until day 10 (Day 10) respectively. **(A)** Cells were harvest and analyzed by flow cytometry for the granularity at three stages (day 0, day6, day10). The cells were initially gated on PI and showed for forward scatter (FSC) and sideward scatter (SSC). Numbers indicate the frequency of cells within indicated areas. The frequencies of indicated cells were compared between each group. Means and standard deviations of cell frequency from three independent analyses are shown. A sample number of 3 patients was used for cytometry assay. *, P < 0.05, **, P < 0.01, NS, not significant (P > 0.05). (B) After incubated with unstimulated condition (control group), LPS, supernatant of HepG2.2.15 cell, and supernatant of HepG2 cells, cultured cells from HD group and CHB group were stained with CD11c (green) and PI (red) and observed under confocal laser-scanning microscope. Representative images from three independent experiments are shown

In order to examine whether CHBaffects the generation ofmo-DCs, we examined the morphology and phenotype of cultured DCs from the HDand patients withCHB. There was no difference between the percentage in HDand HBV patients before generation of mo-DCs (Fig. 1A). After CD14^+^ monocytes were isolated and incubated with recombinant human GM-CSF plus IL-4 for 6 days, the cells were continued to be cultured with unstimulated condition, LPS, supernatant of HepG2 cells and supernatant of HepG2.2.15 cells until day 10. We used flow cytometry to detect the size and granularity of the cultured cells at each time point. We found that the frequency of cells with high forward scatter (FSC) and sideward scatter (SSC) were increased after 6 days cultured with GM-CSF and IL-4 in both HD and HBV group. The cultured cells at day 6 were defined asmo-DCs. The cells from CHB group had a significantly higher frequency of high FSC and SSC cells than that from HD group. After continued cultured with unstimulated condition, LPS, HepG2 cells supernatant to day 10, the cells from CHB group had a comparable frequency of high FSC and SSC cells than that from HD group. However, after cultured with HepG2.2.15 cells supernatant to day 10, the frequency of high FSC and SSC cells from CHB group was significantly higher than that from HD group (Fig. 1A). After cultured with unstimulated condition, LPS, HepG2 cells and HepG2.2.15 cells supernatant to day 10, the cells were stained for CD11c and PI(the nuclei were stained with PI, that represent the viability of the cells). When observed by confocal laser-scanning microscope, all the cells stimulated by LPS, supernatant of HepG2 cells and supernatant of HepG2.2.15 cells from both HD and CHB group were CD11c positive and showed DC-like appearances such as irregular shapes and finger-like projections. Cells cultured in the HepG2.2.15 cells supernatant show bigger size and more projections, and grew more likely in clusters. Comparatively, DCs in unstimulated condition group had a small amount and short projections (Fig. 1B). These results indicated that CD14^+^ monocytes from PBMC of both the HDand patients with CHBcould show the morphology of dendritic cells after exposed to HBV. Furthermore, cells from patients with CHBshowed more granularity and likely higher maturity after exposed HBV.

### Mo-DCs of patients with CHB had the increased capacity to express costimulatory molecules

**Fig. 2:**
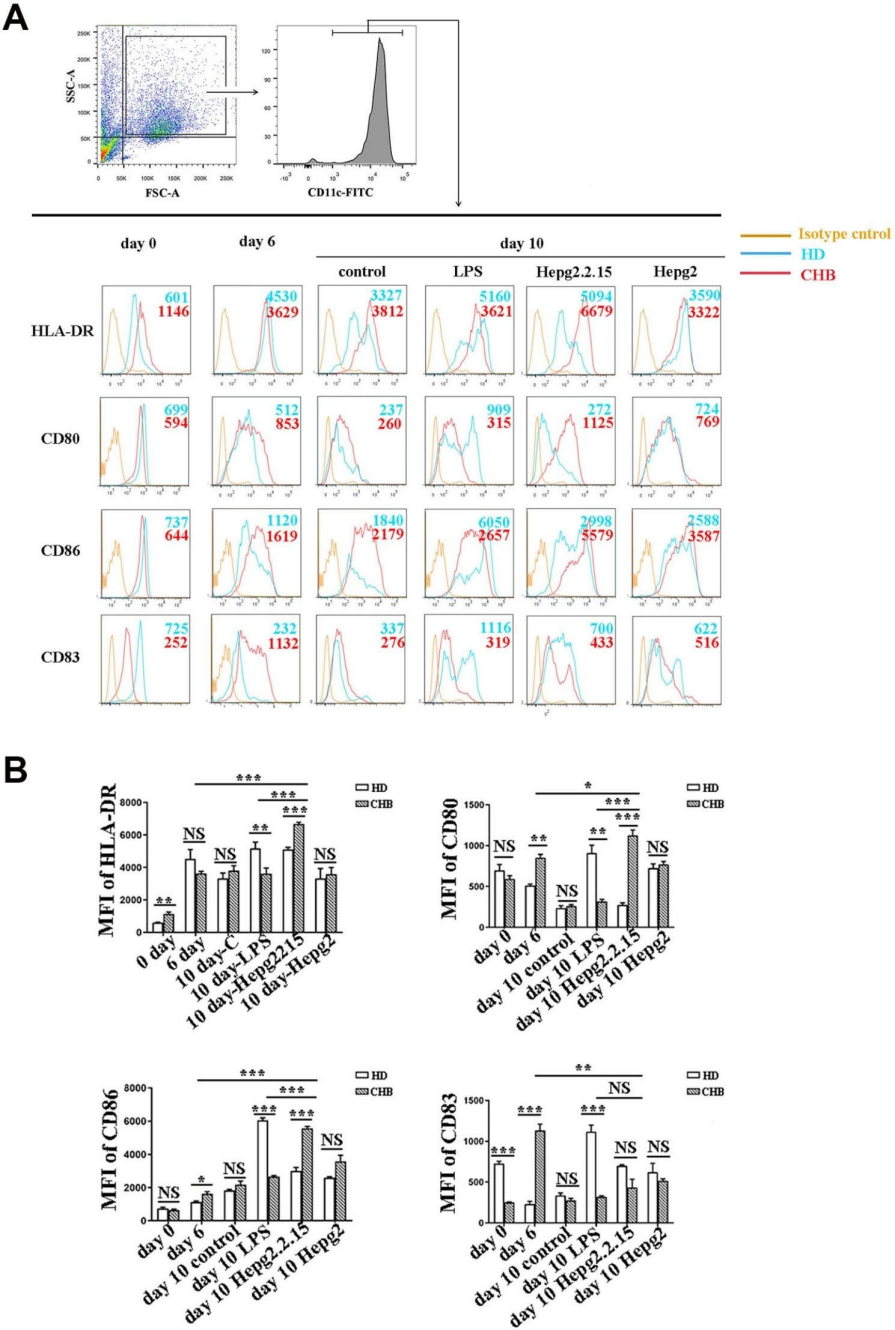
Phenotypic characteristics of mo-DCs. At day 0 (Day 0), CD14^+^ monocytes from HD group and CHB group were isolated and incubated with recombinant human GM-CSF plus IL-4 for 6 days (Day 6). Then the cells were continued to be cultured with unstimulated condition (control group), LPS, supernatant of HepG2.2.15 cells and supernatant of HepG2 cells until day 10 (Day 10) respectively. The cells were stained for CD11c, (human leukocyte antigen- DR )HLA-DR and CD80/CD86/CD83. (A) The living cells were gated initially on CD11c^+^ (upper panel) and then on CD11c^+^HLA-DR^+^ cells (lower three panels). Flow cytometry histograms showing the fluorescence intensities of HLA-DR, CD80, CD86, CD83 of each time point of cells from HD group (red) and CHB group (green) and isotype control (yellow). Numbers from three independent analyses indicated mean fluorescence intensities (MFI). (B) The MFI of HLA-DR in CD11c^+^ cells and CD80/CD86/CD83 in CD11c^+^HLA-DR^+^ cells were compared between each group at each time point. Means and standard deviations of cell numbers from three independent analyses are shown *, P < 0.05, **, P < 0.01, ***, P < 0.001, ****, P < 0.0001, NS, not significant (P > 0.05)

In order to examine whether HBV infection affects the maturation of mo-DCs, we examined the expression of HLA-DR and several co-stimulatory molecules on mo-DCs from HD and CHB (Fig. 2A). After CD14^+^ isolated cells incubated with GM-CSF and IL-4 for 6 days and further cultured with unstimulated condition, LPS, supernatant of HepG2.2.15 cells and supernatant of HepG2 cells until day 10. The freshly isolated cells and cultured cells were stained for PI, CD11c, HLA-DR and CD80/CD86/CD83 and examined by flow cytometry. The cell viability was reflected by the fluorescence intensity (MFI) of PI, and the MFIs of HLA-DR and costimulatory molecules CD80/CD86/CD83 were detected inside life cells gate. The MFI of HLA-DR on freshly isolated CD11c^+^CD14^+^ cells from CHB group are higher than the HD group (p = 0.0011), while the MFI of the costimulatory molecules CD80 and CD86 on CD11c^+^ cells were comparable between CHB group and HD group (Fig. 2A-B). The MFI of HLA-DR on CD11c^+^ cells from CHB group after 10-days cultured in supernatant of HepG2.2.15 cells was increased and even significantly higher than that cultured in LPS. The MFI of HLA-DR on CD11c^+^ cells from CHB group was significantly lower than that from HD group in LPS culture, while higher than that from HD group which cultured in supernatant of HepG2.2.15 cells (Fig. 2A-B). Similarly, the MFI of both CD80 and CD86 on CD11c^+^ cells from CHB group after 10-days cultured in supernatant of HepG2.2.15 cells was increased and even significantly higher than that cultured in LPS. The MFI of both CD80 and CD86 on CD11c^+^ cells from CHB group was significantly lower than that from HD group in LPS culture, but higher than that from HD group which cultured in supernatant of HepG2.2.15 cells (Fig. 2A-B). However, the expression of CD83 showed different trends. The MFI of CD83 on freshly isolated CD11c^+^ cells from CHB group are lower than the HD group. The MFI of CD83 on CD11c^+^ cells from CHB group was significantly lower than that from HD group in LPS culture, while comparable with that from HD group which cultured in supernatant of HepG2.2.15 cells(Fig. 2A-B). These results indicated that CD14^+^ monocytes from PBMC of both health donor and patients with CHBhad increased expression of both MHC II and co-stimulatory molecules and showed a mature DCs phenotypes after exposed to HBV. Furthermore, monocytes cells from patients with CHBappeared to have increased capacity of maturation than that from HDafter re-exposed HBV.

### Secretion of cytokines was enhanced by the mo-DCs from patients with CHB when stimulated by HBV

**Fig. 3:**
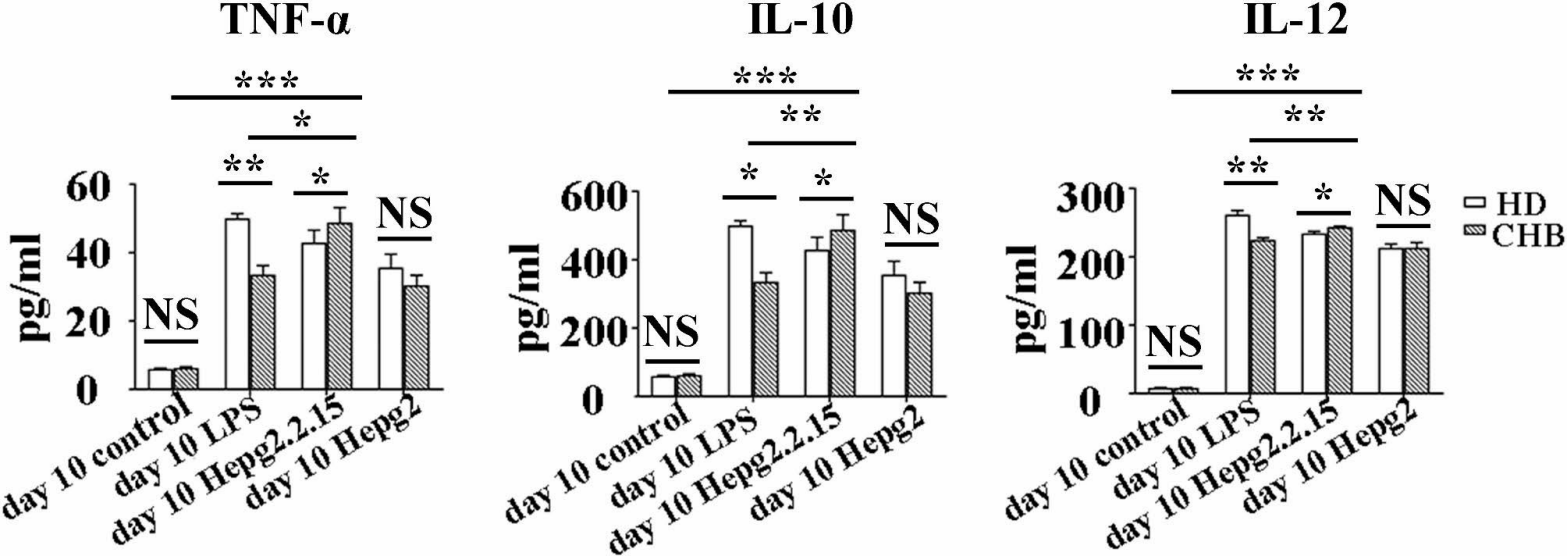
Cytokines secretion of mo-DCs. CD14^+^ monocytes from HD group and CHB group were isolated and incubated with recombinant human GM-CSF plus IL-4 for 6 days. Then the cells were continued to be cultured with unstimulated condition (control group), LPS, supernatant of HepG2.2.15 cells and supernatant of HepG2 cells until day 10 (Day 10) respectively. Then the concentration of tumour necrosis factor-α(TNF-α), IL-10 and IL-12 p70 in the supernatant of cultured cells were detected by enzyme-linked immunosorbent assay (ELISA). The concentration of TNF-α (left panel), IL-10 (middle panel) and IL-12 p70 (right panel), in the supernatant of cultured DCs from indicated group were compared. Means and standard errors of the concentration are shown. Representative results of three independent experiments are shown. *, P < 0.05, **,P < 0.01, ***, P < 0.001, ****, P < 0.0001. NS, not significant

In order to examine whether the function of mo-DCs was affected by HBV infection, we examined the concentration of DC-secretion cytokines, including TNF-α, IL-10 and IL-12 p70, in the supernatant of mo-DCs from HD group and CHB group infection at day 10 after cultured in unstimulated condition, LPS, supernatant of both HepG2.2.15 cells and HepG2 cells by ELISA (Fig. 3). We found that DCs from both HD and CHB group secreted higher level of cytokine TNF-α, IL-10 and IL-12 p70 after stimulated by LPS, supernatant of both HepG2.2.15 cells and HepG2 cells than that with unstimulated condition. The amount of cytokine TNF-α, IL-10 and IL-12 p70 secreted by mo-DCs from both HD and HBV group after stimulated by supernatant of HepG2 cells was significantly lower than that by LPS and supernatant of HepG2.2.15 cells. The mo-DCs from HD after cultured with LPS secreted higher amount of all cytokines that we examined, including TNF-α, IL-10 and IL-12 p70 than that with supernatant of HepG2.2.15 cells and supernatant of HepG2 cells. However, the amount of cytokines, including TNF-α, IL-10 and IL-12 p70 secreted by mo-DCs from CHB group cultured with supernatant of HepG2.2.15 cells was significantly higher than that with LPS. Furthermore, the secretion of all the cytokines we examined by mo-DCs from CHB group was significantly higher than that from HD group after stimulated with HBV. These results indicated that the DCs from patients with CHBhad the enhanced capacity of cytokine secretion when re-stimulated by HBV.

### Enhanced autophagy in DCs was induced by HBV infection

**Fig. 4:**
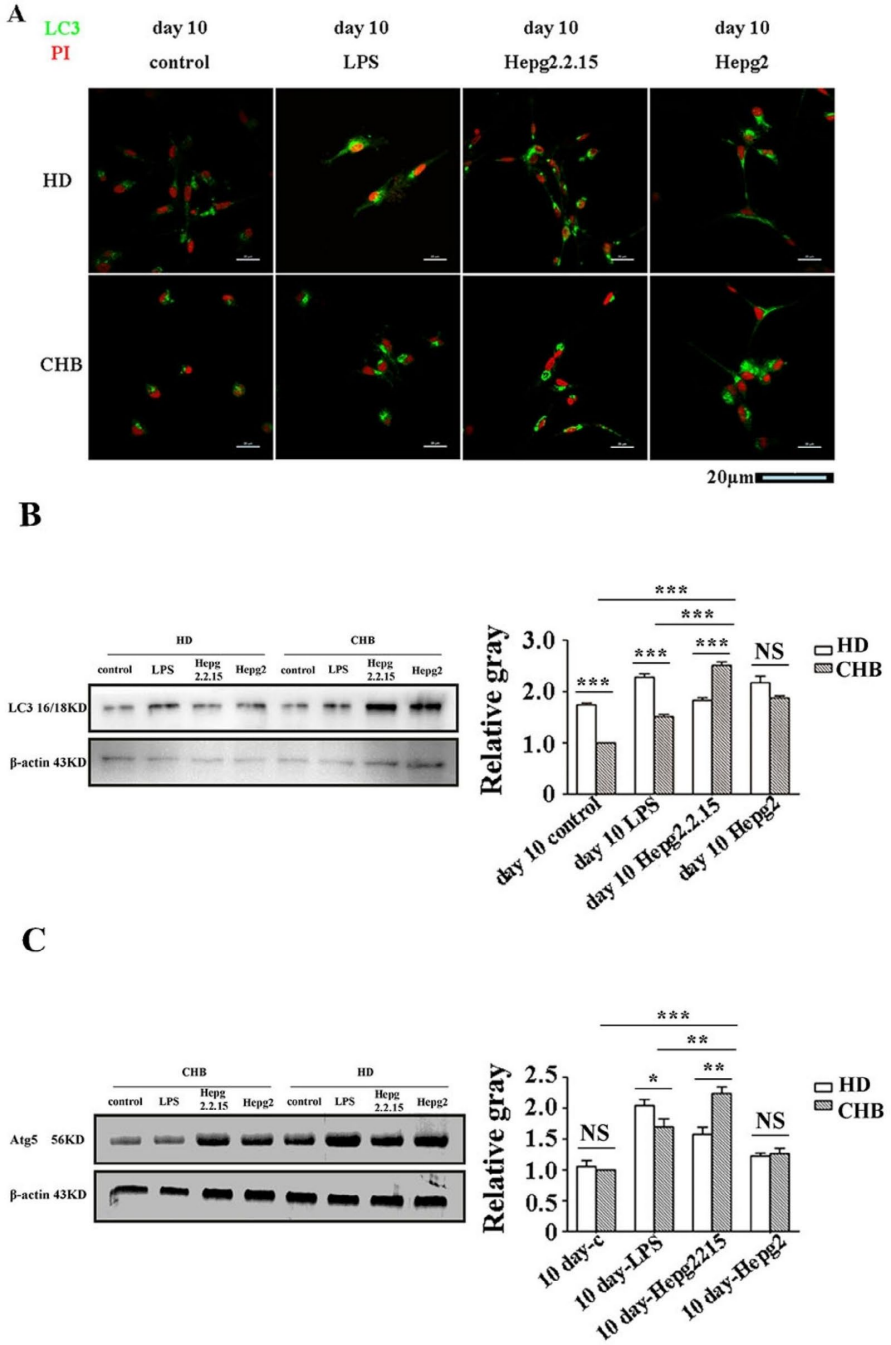
Expression of autophagy-related proteins in DCs. Two-color immunofluorescence analysis of DCs from HD (upper panel) and patients with CHB(lower panel), which cultured with control medium, LPS, supernatant of HepG2.2.15 cells, supernatant of HepG2 cells respectively. Cultured cells were stained with anti-LC3 antibody (green) and PI (red). Representative images from three independent experiments are shown. **(B-C)** Western blot analysis of associated protein 1 light chain (LC3 protein) **(B)** and autophagy-related protein-5(Atg5) protein **(C)** expression in cultured DCs from HD and patients with CHB. Shown are the representative data of three independent experiments. Means and standard deviations of relative grey-scale intensity of LC3 (B, right graph) and Atg5 (C, right graph) expression in indicated group are shown. *, P < 0.05, **, P < 0.01, NS, not significant (P > 0.05)

Since the MHC II expression and cytokine secretion was increased in mo-DCs from patients with CHBwhen re-boomed by HBV, we preclude that the autophagy was also affected by the HBV infection. The expression of microtubule-associated LC3 protein which is important to the autophagy in DCs was examined. Immunofluorescent analysis showed that the LC3 in the mo-DCs from HDafter stimulated by LPS was stronger than that after stimulated by supernatant of HepG2 cells and supernatant of HepG2.2.15 cells (Fig. 4A). However, in the mo-DCs from patients with CHB, the LC3 granularity expression after stimulated by supernatant of HepG2.2.15 cells was strongest and most agminated among the three treated group, which include LPS, HepG2.2.15 and HepG2 supernatant. The LC3 expression in the mo-DCs from patients with CHB also showed strongest fluorescence intensity after stimulated by supernatant of HepG2.2.15 cells among the three treated group (Fig. 4A).Western blot analysis showed that the expression of both LC3 protein by DCs from HD was significantly higher than that from patients with CHBafter stimulated by LPS (Fig. 4B). The expression of LC3 protein by DCs from HD was comparable with that from patients with CHBafter stimulated by supernatant of HepG2 cells. However, the expression of LC3 protein by DCs from patients with CHBwas significantly higher than that from HD after stimulated by supernatant of HepG2.2.15 cells which contained HBV (Fig. 4B). The expression of autophagy-related protein5(Atg5), a key protein which was involved in the positive regulation of autophagy, in DCs were examined. The similar results showed that the expression of Atg5 protein by DCs from HD was significantly higher than that from and patients with CHBafter stimulated by LPS (Fig. 4C). The expression of Atg5 protein by DCs from HD was comparable with that from patients with CHBafter stimulated by supernatant of HepG2 cells. The expression of Atg5 protein by DCs from patients with CHBwas significantly higher than that from HD after stimulated by supernatant of HepG2.2.15 cells which contained HBV (Fig. 4B). These results indicated that the autophagy in DCs was enhanced by the infection of HBV.

## Discussion

In this study, we found that the expression of both MHC II molecules and co-stimulated molecules including CD80, CD86 in the mo-DCs from patients with CHBwas significantly higher than that from HDwhen cultured with supernatant of HepG2.2.15 cells. The amount of cytokines, including TNF-α, IL-10 and IL-12, secreted by mo-DCs from patients with CHBwas also significantly higher than that from HD when stimulate by HBV. Interestingly, the expression level of autophagy-related proteins including Atg5 and LC3 in DCs from patients with CHBwas significantly increased when compared with that from HDwhen re-exposed to HBV.

DCs are known as the most potent professional APC in the body which are important for T cell stimulation. Several researches showed immature phenotype and defective function of DCs during HBV infection are associated with the ineffectively induction of adaptive immune responses for the clearance of HBV(10–13). However, there are several other researches showed that the frequency and phenotype of DC were comparable between patients with CHBand HD(14, 15). Whether DCs in chronic HBV patients are phenotypically and functionally equal to DCs from HDhas been a longstanding point of debate. Mo-DCsare considered to mimic the inflammatory DC population that differentiate during periods of inflammation and are capable of promoting T-cell development and antigen processing (16). In this research, we obtained mo-DCs derived from CD14 + monocytes of patients with CHBandHD. After 6 days of in vitro culture with GM-CSF and IL-4, these cells from both the HD and patients with CHBcould show the morphology of dendritic cells after exposed to HBV. Furthermore, cells from patients with CHBshowed more granularity and likely higher maturity after exposed HBV (Fig. 1). These results indicated that CD14 + monocytes of patients with CHBhave the similar ability to differentiate into DCs with that fromHD.

After dendritic cells capture invading pathogen, they gradually become mature. Mature DCs stimulate T lymphocytes for activation and differentiation and then initiate immune responses through the expression of MHC, co-stimulatory molecules including CD80, CD83, CD86, various cluster of differentiation and the production of cytokines, including TNF-α, IL-10 and IL-12 [25, 26]. Several studies shared a conclusion that the expression of co-stimulatory molecules was lower on immature mo-DC [10–13] from HBV-infected individuals than from healthy individuals; however, the expression recovered to the level of healthy donor mo-DC following maturation. The different results may be from different patients and different maturation stimuli. In study by Beckebaum et al. [10], they included the patients with cirrhosis, while in study by Tavakoli et al. [14], they brought both active HBV patients and inactive carriers into research. In our research, we choose patients with CHBand normal liver function but without fibrosis. CD14^+^ monocytes from PBMC of both health donor and patients with CHBhad increased expression of both MHC II and co-stimulatory molecules and showed a mature DCs phenotypes after exposed to HBV. Furthermore, monocytes cells from patients with CHBappeared to have increased expression of MHC II and co-stimulatory molecules, including CD80 and CD86, but not CD83 than that from HDafter stimulated by HBV. Our results indicated that mo-DCs from patients with CHBhave increased capacity of maturation than that from HDafter re-exposed HBV.

TNF-α can act as an autocrine growth factor for dendritic cell-induced T-cell proliferation [27]. Besides, it plays an essential role in the maturation of DC. Van der Molen et al. have demonstrated that addition of exogenous TNF-α induced maturation of DC [4]. Secretion of IL-12 by DCs is critical for the induction of Th1 differentiation, as well as for the proliferation and enhanced cytotoxic activity of natural killer cells [28]. IL-10, a predominant Th2-type cytokine, plays a role in immune suppression and has counter-regulatory properties to a number of pro-inflammatory cytokines, including IL-12 [29]. Also, it can induce membrane expression of MHC II molecules on macrophages [30]. Similar to the phenotype experiments, most studies have found that cytokine production of DCs was impaired following exposure to HBV or HBV antigens [31]. In our research, supernatant of HepG2.2.15 cells could stimulate mo-DCs from both HD and HBV patients with CHBto secrete higher level of cytokine TNF-α, IL-10 and IL-12 p70 than LPS. Furthermore, we found that the production of TNF-α, IL-10 and IL-12 p70 mo-DCs from CHB group was significantly higher than that from HD group after stimulated by the supernatant of HepG2.2.15 cells. Our results suggested that the mo-DCs from patients with CHBnot only showed maturation phenotype, but also had the enhanced capacity of cytokine secretion when re-stimulated by HBV.

Inducing maturation of DCs enhance the autophagic process, increases antigen processing and upregulates costimulatory molecules on the surface of mo-DCs. Related to Atg, including Atg5 and LC3, the phagosome ultimately fuses with lysosomes to form phagolysosomes, through which the contents of the phagosomes, including degraded proteins, can interact with the MHC II molecules [32]. Li et al. report that HBV can enhance the autophagic process in hepatoma cells without promoting protein degradation by the lysosome, and the autophagy machinery is needed for HBV production and envelopment [33]. Whether the HBV infection affect the autophagy machinery of DCs and whether the modified phenotype and function of DCs is associated with the autophagy pathway are still unclear. Microtubule-associated LC3 protein in the mo-DCs from patients with both CHBand HD was significantly granularity expressed and agminated after stimulated by HBV. We also demonstrated that the expression of Atg5 and LC3 in the mo-DCs from patients with CHBis enhanced after stimulated by the HBV. These results indicated that in vitro HBV could enhance autophagy of mo-DCs from HBV patients and health donors. The enhanced autophagy pathway of mo-DCs in the presence of HBV-stimulation in vitro is exactly in accordance with the increased expression of co-stimulated molecules CD80, CD86 and the increased production of cytokines including TNF-α, IL-10 and IL-12 p70. Our results suggested that HBV-stimulation was able to enhance the expression of co-stimulatory molecules (CD80 and CD86) of the mo-DCs, increase the cytokine secretion, and also enhance autophagy from both normal donors and patients withCHB. However, the opposite effect was observed in response to LPS, regarding DC maturation profile, cytokines production and autophagy. The reason may be that chronic infection affects the response of DCs to LPS (anergy effect).

Our study was limited in the small sample size, a larger number of cases would be better to validate the result. In the future, we will include more individuals to support the conclusion.

## Conclusions

In conclusion, the present results indicated that DCs from patients with CHBcould intactly even intensively present antigen and express surface molecules. The increased activation of DCs might be related to the enhanced autophagy of DCs in HBV infection. However, the correlation between enhanced function and autophagy state of DCs in HBV infection needs to be further investigated.

### Electronic supplementary material

Below is the link to the electronic supplementary material.

Supplementary Material 1

Supplementary Material 2

Supplementary Material 3

Supplementary Material 4

Supplementary Material 5

Supplementary Material 6

Supplementary Material 7

Supplementary Material 8

Supplementary Material 9

Supplementary Material 10

## Data Availability

The datasets used and/or analysed during the current study are available from the corresponding author on reasonable request.

## Abbreviations

APCs: antigen-presenting cells
ALT: alanine aminotransferase
AST: aspartate aminotransferase
Atg: autophagy-related protein
CD: cluster of differentiation antigen
CHB: chronic HBV infection
CTL: cytotoxic T-cell
ELISA: enzyme-linked immunosorbent assay
FSC: forward scatter
GM-CSF: granulocyte-macrophage colony-stimulating factor
HAV: hepatitis A virus
HBV: hepatitis B virus
HBsAg: hepatitis B virus surface antigen
HCC: hepatocellular carcinoma
HCV: hepatitis C virus
HD: healthy donor(s)
HEV: hepatitis E virus
HIV: human immunodeficiency virus
HLA-DR: human leukocyte antigen DR
IL: interleukin
LC3: associated protein 1 light chain
LPS: lipopolysaccharide
MFI: mean fluorescence intensity
MHC: major histocompatibility complex
mo-DCs: monocyte-derived dendritic cells
PBMCs: peripheral blood mononuclear cells
SSC: sideward scatter
TNF-α: tumor necrosis factor-α
