# Supplementary material for: Function and autophagy of monocyte-derived dendritic cells is affected by hepatitis B virus infection
Source: BMC Immunol. 2023 Sep 26;24:31. doi: 10.1186/s12865-023-00571-2 (PMC10521579; doi:10.1186/s12865-023-00571-2)
Supplement: Supplementary file 1 — Supplementary Material 1 [file 12865_2023_571_MOESM1_ESM.docx]

supplementary table1：patient characteristics

| characteristic | healthy blood donor（=3） | patients with chronic HBV infection（=3） | p |
| --- | --- | --- | --- |
| Age (years) | 55.33±1.53 | 55.67±2.52 | 0.85 |
| HBV DNA concentration（IU/ml） | 286000±187158 | 344333±231897 | 0.752 |
| ALT（ U/L） | 31.33±5.03 | 29±8.54 | 0.704 |
| AST（ U/L） | 22.33±5.13 | 17.33±3.79 | 0.246 |
