# Supplementary material for: Function and autophagy of monocyte-derived dendritic cells is affected by hepatitis B virus infection
Source: BMC Immunol. 2023 Sep 26;24:31. doi: 10.1186/s12865-023-00571-2 (PMC10521579; doi:10.1186/s12865-023-00571-2)
Supplement: Supplementary file 2 — Supplementary Material 2 [file 12865_2023_571_MOESM2_ESM.docx]

**Supplementary Figure legends: Expression of autophagy-related proteins in DCs**

supFig1 Original images of Western blot analysis of LC3 protein in cultured DCs from health donors (HD) and patients with chronic HBV infection (CHB)

supFig2 Original images of Western blot analysis of β-actin protein of LC3 in cultured DCs from health donors (HD) and patients with chronic HBV infection (CHB)

supFig3 Multiple exposure image-1 of Western blot analysis Atg5 protein in cultured DCs from health donors (HD) and patients with chronic HBV infection (CHB)

supFig4 Multiple exposure image-2 of Western blot analysis Atg5 protein in cultured DCs from health donors (HD) and patients with chronic HBV infection (CHB)

supFig5 Multiple exposure image-3 of Western blot analysis Atg5 protein in cultured DCs from health donors (HD) and patients with chronic HBV infection (CHB)

supFig6 Multiple exposure image-1 of Western blot analysis β-actin protein of Atg5 in cultured DCs from health donors (HD) and patients with chronic HBV infection (CHB)

supFig7 Multiple exposure image-2 of Western blot analysis β-actin protein of Atg5 in cultured DCs from health donors (HD) and patients with chronic HBV infection (CHB)

supFig8 Multiple exposure image-3 of Western blot analysis β-actin protein of Atg5 in cultured DCs from health donors (HD) and patients with chronic HBV infection (CHB)
