# Supplementary figures and images for: Function and autophagy of monocyte-derived dendritic cells is affected by hepatitis B virus infection
Source: BMC Immunol. 2023 Sep 26;24:31. doi: 10.1186/s12865-023-00571-2 (PMC10521579; doi:10.1186/s12865-023-00571-2)

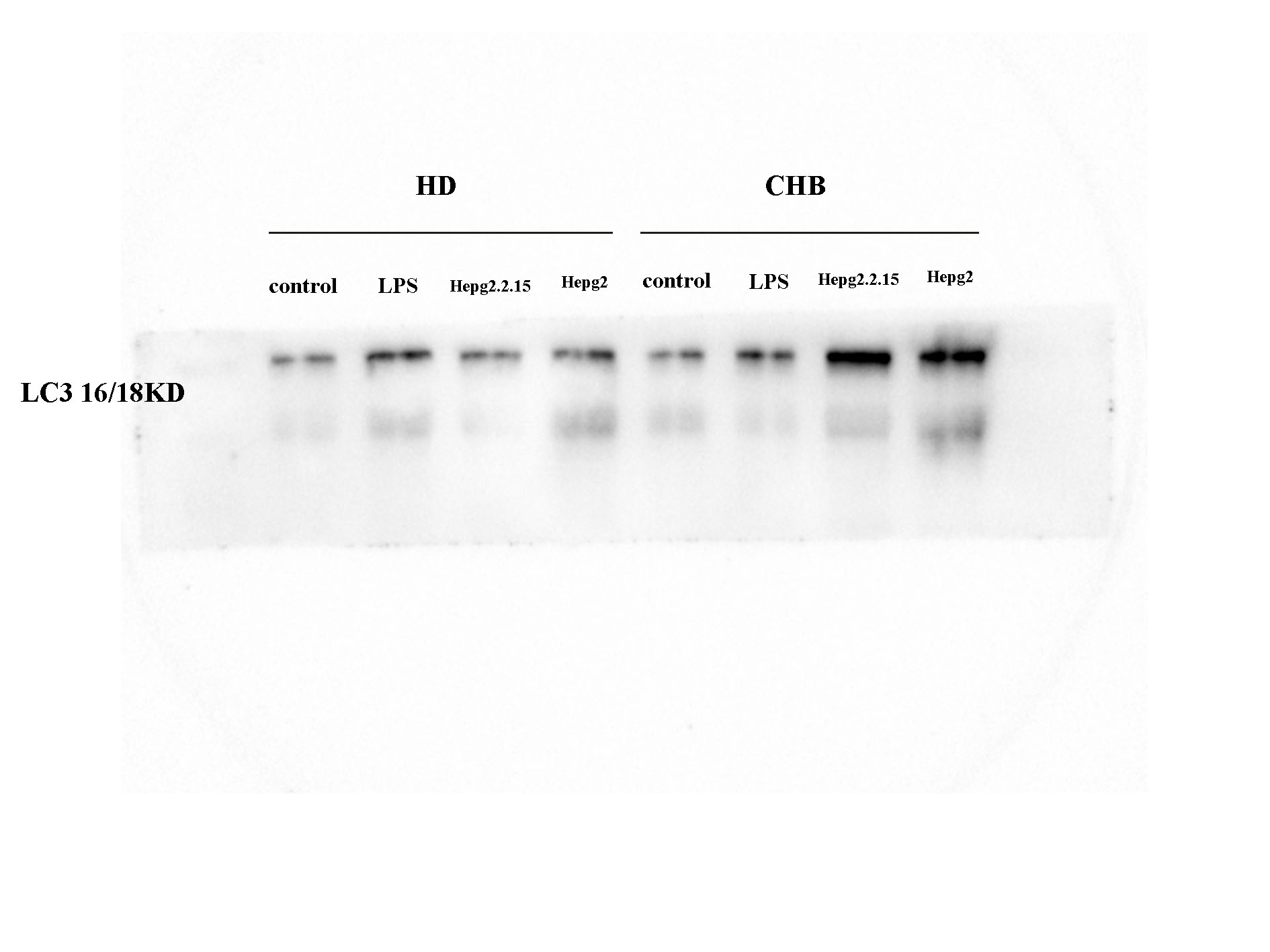

Supplement: Supplementary file 3 — Supplementary Material 3 [file 12865_2023_571_MOESM3_ESM.jpg]

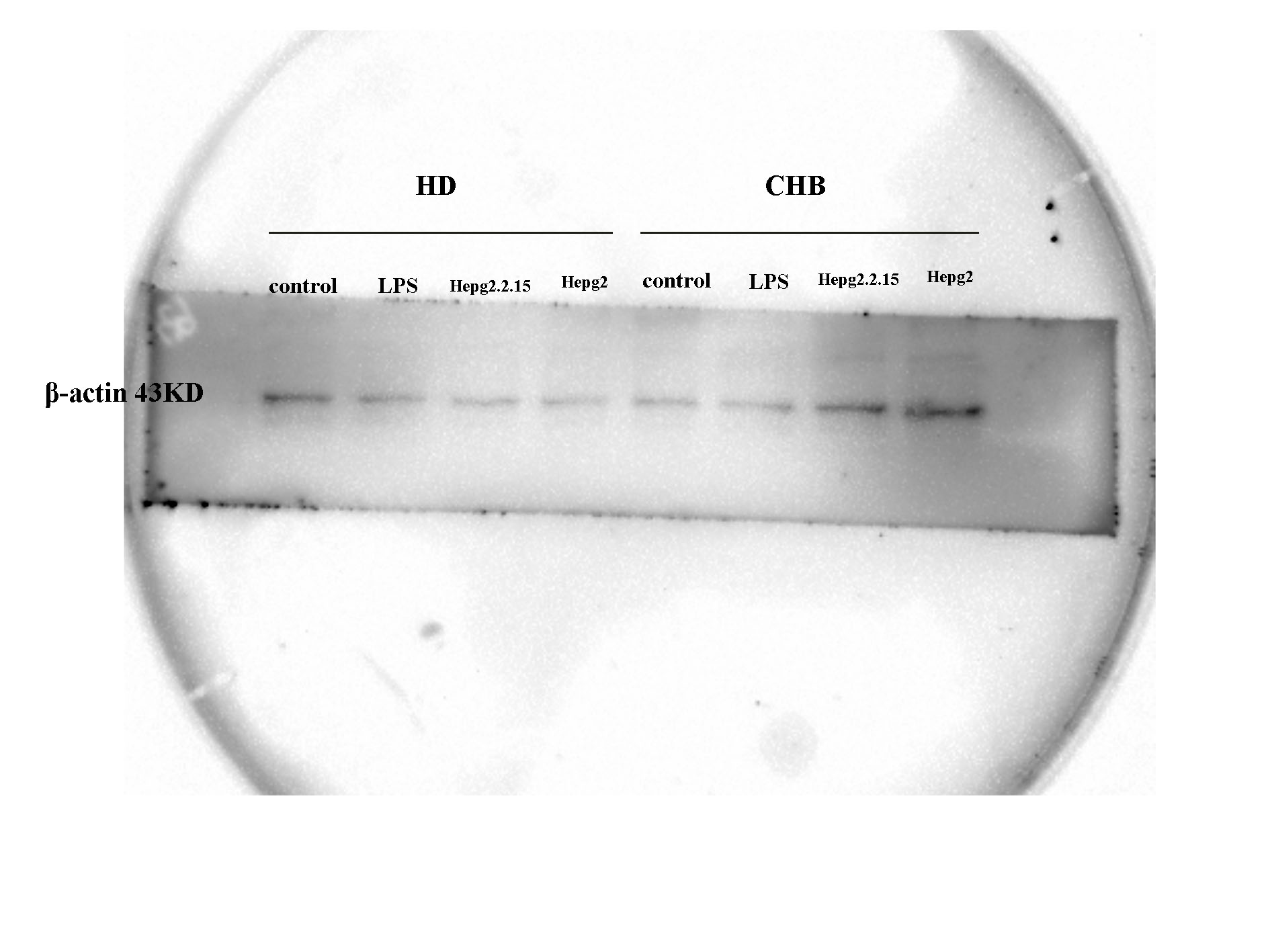

Supplement: Supplementary file 4 — Supplementary Material 4 [file 12865_2023_571_MOESM4_ESM.jpg]

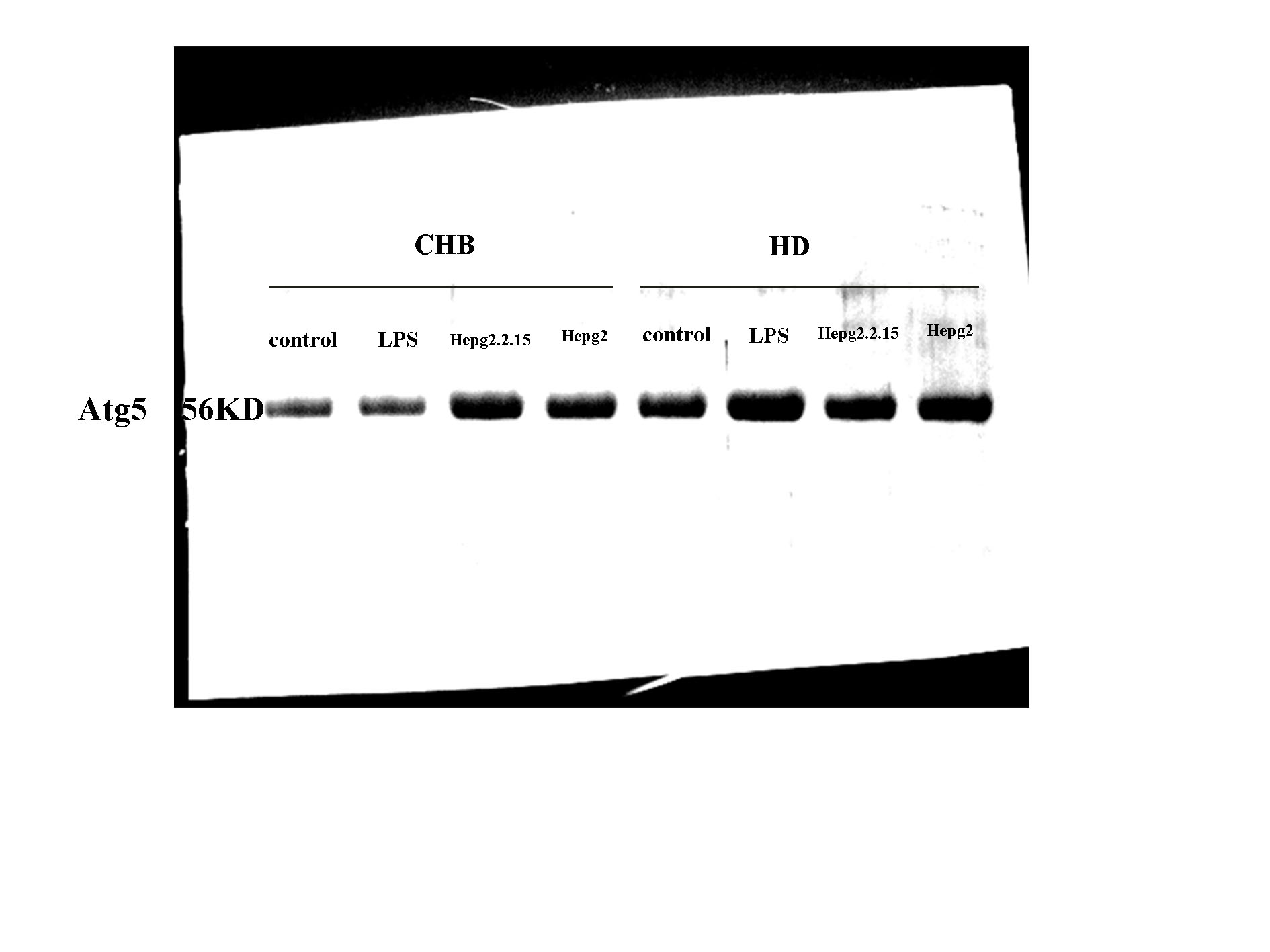

Supplement: Supplementary file 5 — Supplementary Material 5 [file 12865_2023_571_MOESM5_ESM.jpg]

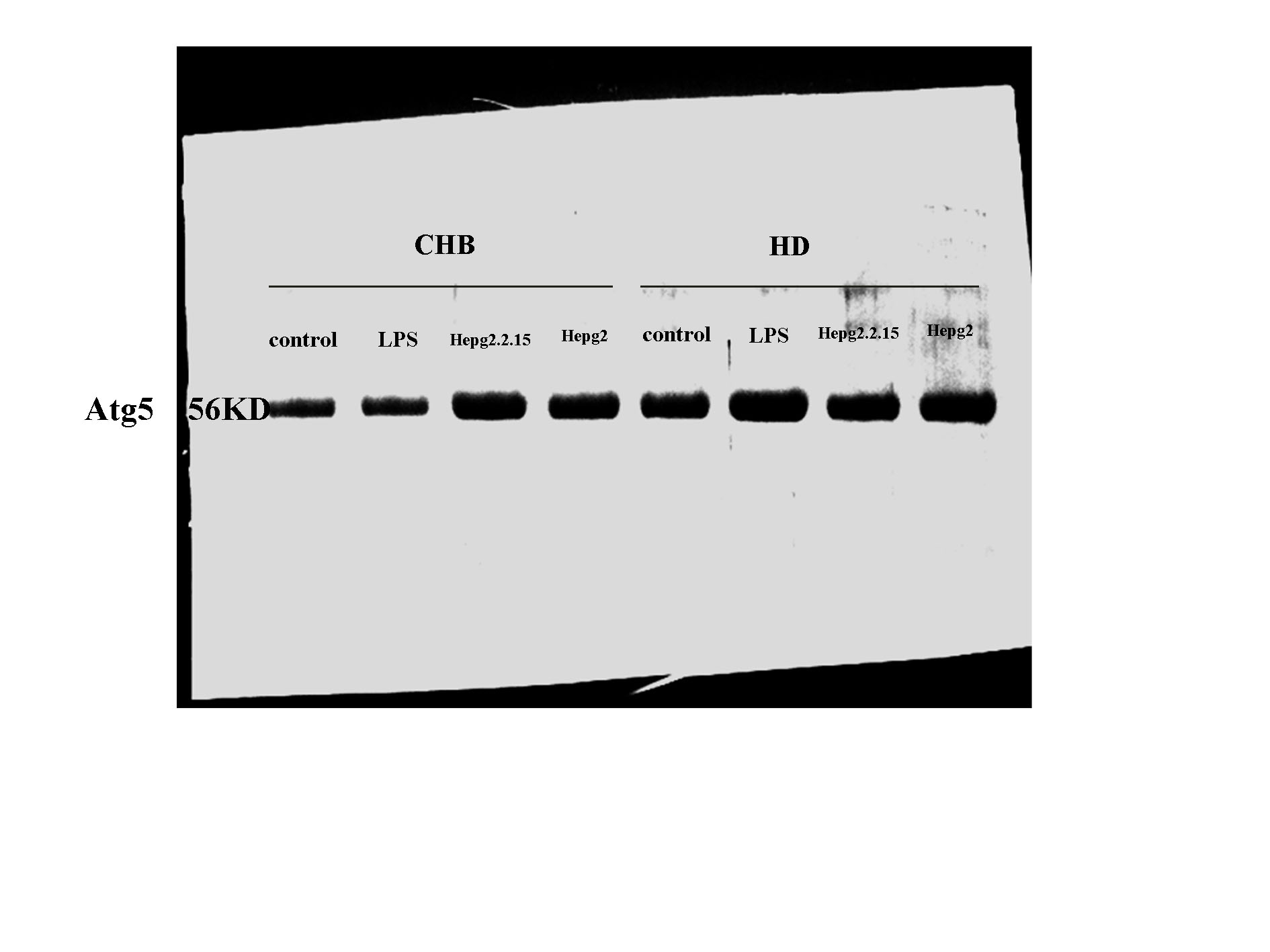

Supplement: Supplementary file 6 — Supplementary Material 6 [file 12865_2023_571_MOESM6_ESM.jpg]

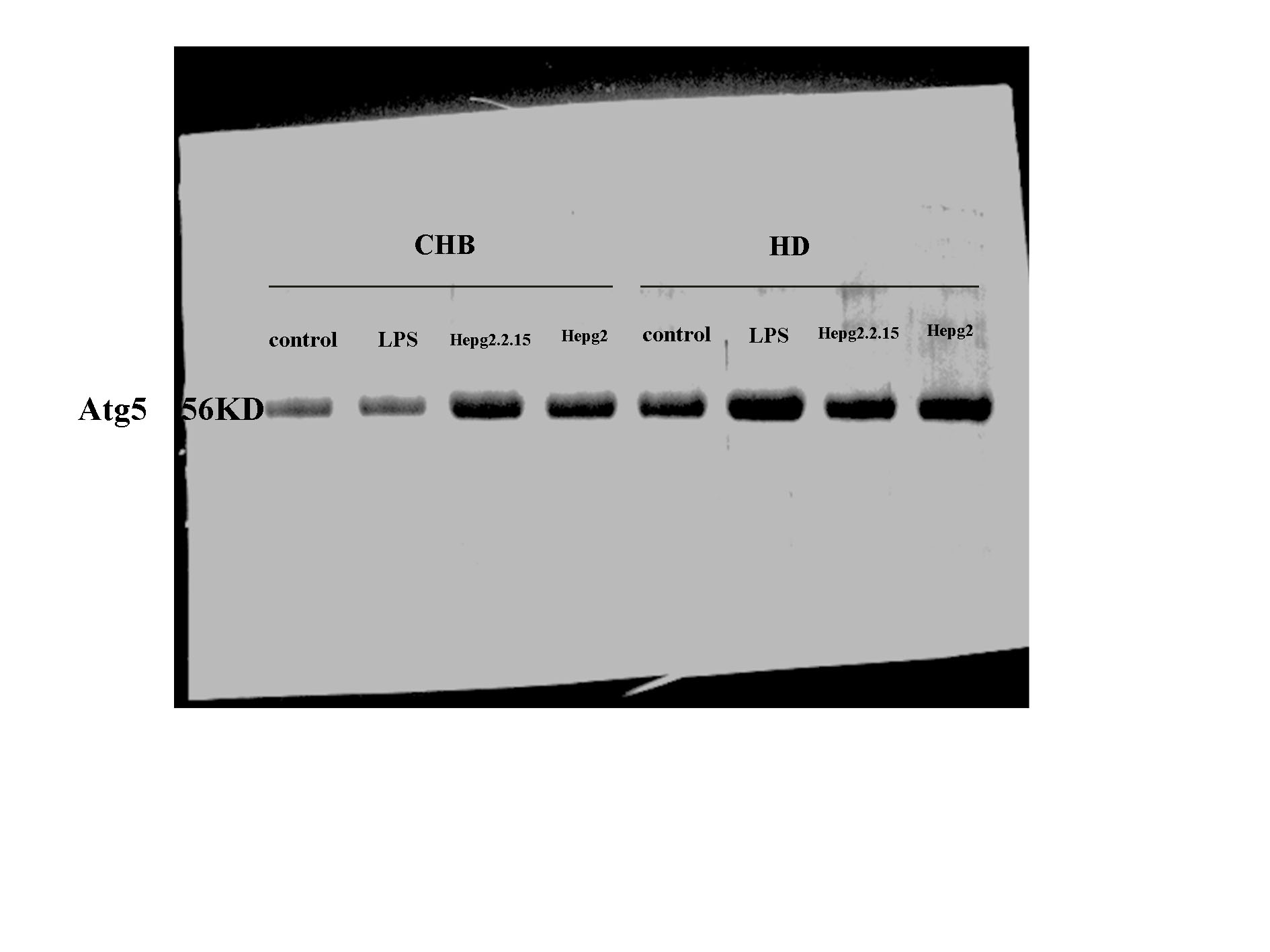

Supplement: Supplementary file 7 — Supplementary Material 7 [file 12865_2023_571_MOESM7_ESM.jpg]

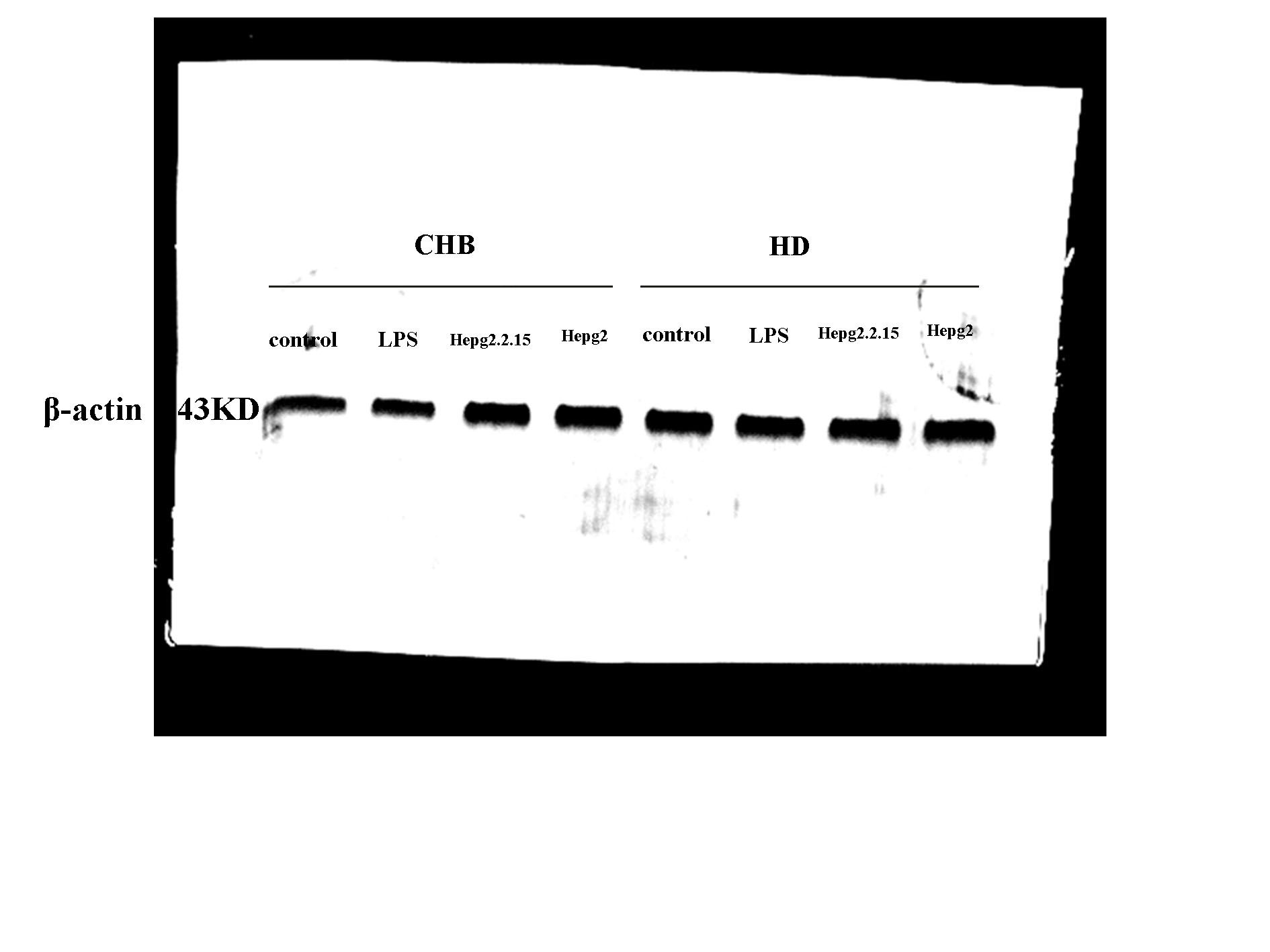

Supplement: Supplementary file 8 — Supplementary Material 8 [file 12865_2023_571_MOESM8_ESM.jpg]

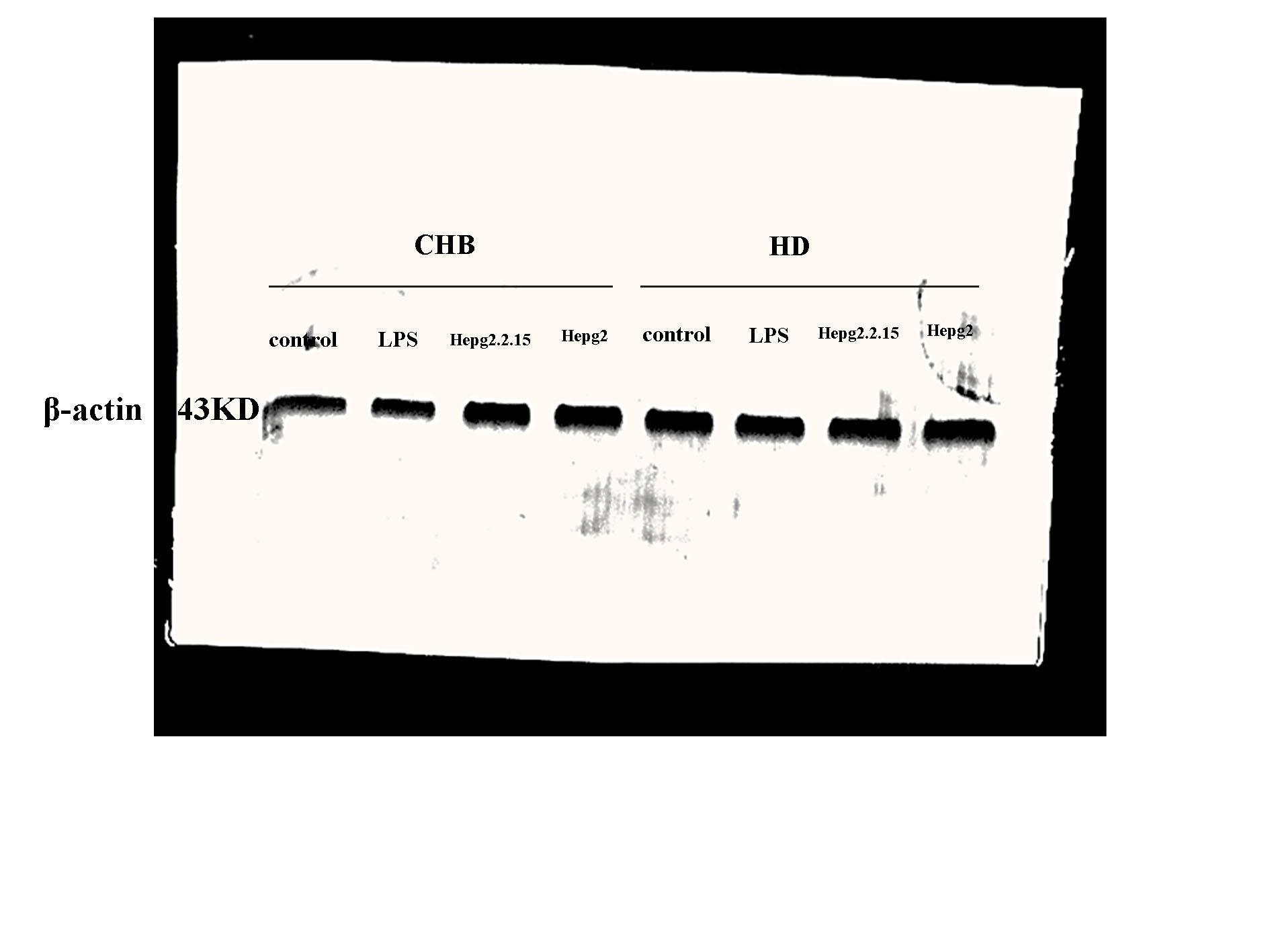

Supplement: Supplementary file 9 — Supplementary Material 9 [file 12865_2023_571_MOESM9_ESM.jpg]

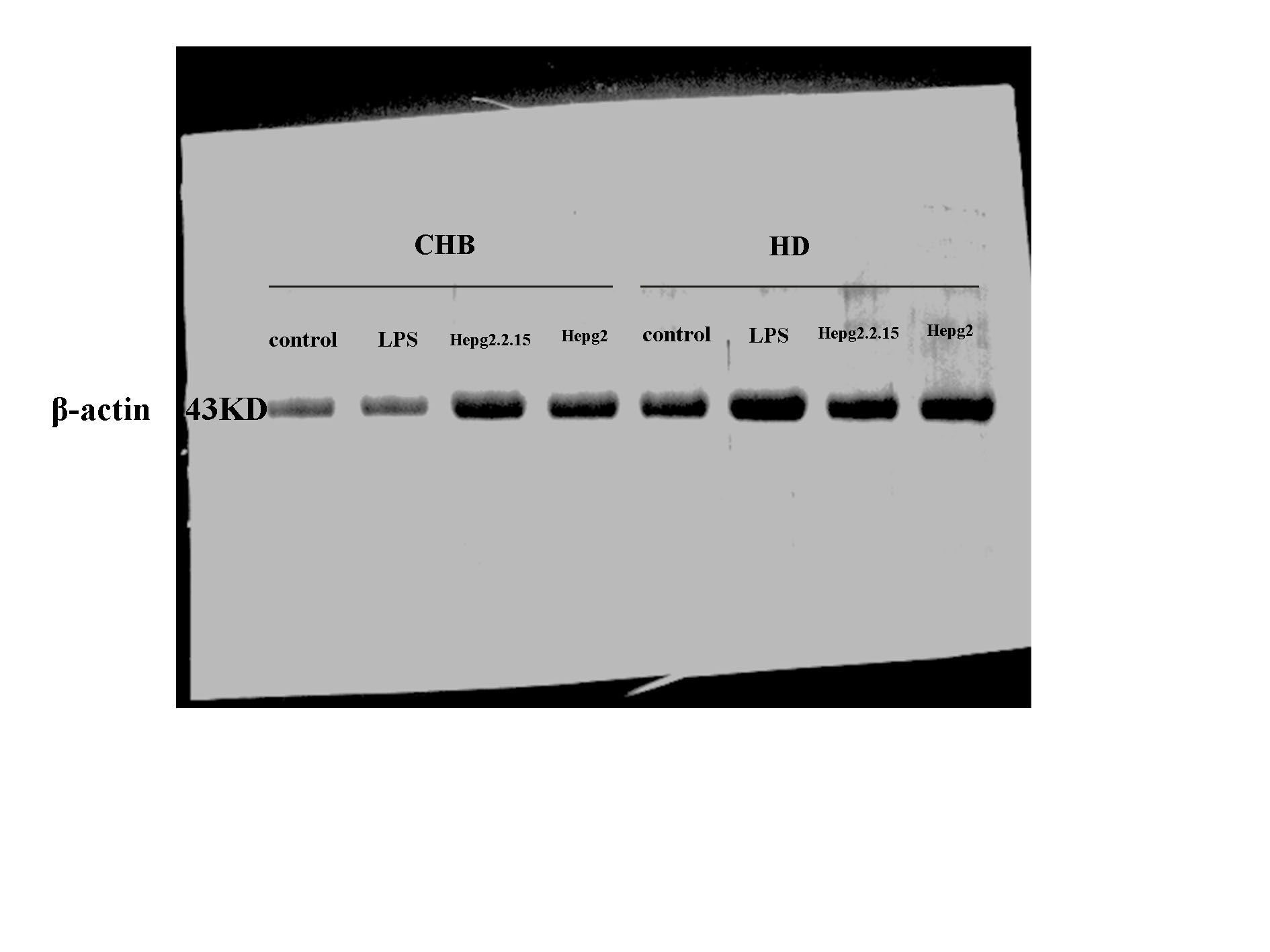

Supplement: Supplementary file 10 — Supplementary Material 10 [file 12865_2023_571_MOESM10_ESM.jpg]
